# Cholecystectomy or Cholelithiasis – a Missed Marker for Hyperlipidaemia? A Combined Retrospective and Prospective Study

**DOI:** 10.4021/gr2008.11.1246

**Published:** 2008-11-20

**Authors:** Sivakumaran Sabanathan, Soonita Oomeer, Lloyd R Jenkinson

**Affiliations:** a Department of Surgery, North West Wales NHS Trust, Penrhosgarnedd, Bangor LL57 2PW, UK

**Keywords:** cholecystectomy, hyperlipidaemia, gallstones hypercholesterolaemia, hypertri-glyceridaemia

## Abstract

**Background:**

Multiple studies have shown an association between gallstones and abnormal lipids and the latter increases the risk of coronary artery disease and stroke. Our study investigates the current incidence of lipid abnormalities in patients who have undergone cholecystectomy (retrospective study) and who have gallstones (prospective study).

**Methods:**

We conducted a retrospective analysis of the lipid profiles of 715 patients who underwent cholecystectomy in a rural district general hospital from 2003 to 2006. Details of the cholecystectomy patients were obtained from Patient Information and Management System (PIMS) and cross-referenced with biochemical and histological databases. Following this a prospective study was undertaken of 129 patients presenting with gallstones.

**Results:**

Of the 715 patients, three quarters were women. Only 36.2% of women and 36.9% of men who had a cholecystectomy had a full lipid profile including high density lipoproteins (HDL) and low density lipoproteins (LDL). Of these, 76.4% of women and 70.7% of men had an abnormal lipid profile. In the prospective group, 91.1 % of women and 96.3 % of men had a full lipid profile. These were abnormal in 81.4% of women and 70.4 % of men. Hypercholesterolemia and raised LDL were the most common abnormalities in both sexes. Hypertriglyceridaemia was common in both sexes in both the groups.

**Conclusions:**

Patients who have had a cholecystectomy or gallstones should have a full fasting lipid profile, including HDL and LDL, as a large proportion will be abnormal. Current guidelines suggest they are at an increased risk of cardiovascular disease and should be treated.

## Introduction

Patients presenting with renal stones are fully assessed for an underlying metabolic disorder but this is not established practice for patients with gallstones. Evidence from over 30 years ago showed that over half of patients with gallstones would have a lipid disorder[1]. This would increase their risk of developing coronary heart disease and stroke [2-8]. The current national service framework for coronary artery disease recommends a cholesterol level < 5 mmol/L and low-density lipoprotein (LDL) < 3 mmol/L in patients with 30% risk of developing coronary heart disease over ten years[9]. In the United Kingdom around 5.5 million people have gallstones and over 50, 000 cholecystectomies are performed each year suggesting there is a large population at risk[10].

Recent European studies have shown that hypertriglyceridaemia and low levels of high-density lipoprotein cholesterol (HDL), in addition to hypercholestrolaemia, are common findings[11, 12], which in turn are considered risk factors for coronary artery disease and stroke[13-15].

There is no current up to date UK data on which to base our current practice. This study retrospectively investigates the frequency of lipid disorders in patients who had undergone cholecystectomy and prospectively on patients with cholelithiasis to identity the proportion at risk of coronary heart disease or stroke.

## Methods

An initial retrospective study of patient who had a cholecystectomy was performed with a subsequent prospective analysis of patients with proven cholelithiasis.

We obtained the details of all patients who had a cholecystectomy at the North West Wales NHS Trust from April 2003 to May 2006 inclusive from Patient Information and Management System (PIMS). The available data was cross-referenced with the biochemical and histological database (Telepath) to identify those patients who had a cholecystectomy and a lipid profile. We have previously validated the hospital PIMS in surgical patients and demonstrated an error rate of about 5%[16]. The initial analysis was done using the hospital number but a second check was made for General Practitioners (GP) requests using the date of birth. The basic lipid profile reported by our laboratory was cholesterol and triglycerides. HDL and LDL were analysed only if requested and if the sample was fasting.

Our hospital does not have a radiological database and we therefore, could not retrospectively identify patients with gallstones on ultrasound scan. There was a high incidence of lipid abnormality in the retrospective study but the percentage of patients who had a full lipid profile was low. We then conducted a prospective study of patients with proven gallstones referred to a single Upper Gastro-intestinal surgeon either as an outpatient or as an emergency to give a more accurate assessment of the lipid abnormalities.

We analysed the patient demographics, the serum cholesterol, triglycerides, HDL cholesterol and lLDL cholesterol. The source of the request and whether it was done pre-operatively were recorded. Chi-square was used to analyse the differences between the sexes.

The study was reviewed and cleared by the North West Wales Ethical committee board at Bangor.

## Results

### Retrospective study

Of the 715 cholecystectomies which were performed, 536 (75%) were females and 179 were (25 %) males. The median age of the females was 55 years (range 15-90) and 61 years (29-88) for males.

Only 194/536 (36.2%) of women and 66/179 (36.9%) of men had a full lipid profile including HDL and LDL. A partial lipid profile (cholesterol and triglyceride) was done in 119/536 (21.8%) of women and 57/179 (31.8%) of men. 76.4% of women and 70.7% of men had one or more abnormalities. The lipid profiles were done pre-operatively in 247/313 (78.9%) of women and 111/123 (90.2%) of men. This was done by the GP in 334/436 (76.6%).

Using the current NSF guidelines, 211/313 (67.4%) of women and 66/123 (53.7%) of men had a cholesterol > 5 mmol/L, and 121/194 (62.4%) of women and 37/66 (56.1%) of men had an LDL >3 mmol/L. Full details are shown in table 1.

**Table 1:** Retrospective study of lipid profiles in patients who have had a cholecystectomy

Lipids (abnormal range)	Females	Males	P value *
Cholesterol (> 5.0 mmol/L)	211/313 (67.4%)	66/ 123 (53.7%)	≤ 0.01
Triglycerides (> 1.92 mmol/L)	108/313 (34.5%)	50/ 123 (40.7%)	ns
HDL cholesterol (< 1.00 mmol/L)	9/199 (4.5%)	13/72 (18.1%)	≤ 0.001
LDL cholesterol (> 3.00 mmol/L)	121/194 (62.4%)	37/66 (56.1%)	ns

* Chi square with 1 degree of freedom. ns = not significant.

### Prospective study

The 129 patients with cholelithiasis were studied, of which 102 (79%) were females and 27 (21%) were males. The median age of females was 49 (range 18-88) and 59 (range 26-77) for males. 91.1% (93/102) of women and 96.3% (26/27) of men had a full lipid profile including HDL and LDL. Most of the tests were done in the hospital if the patients were fasted or a request sent to the GP.

Overall 81.4% of women and 70.4 % of men had abnormal lipid profiles. 66/102 (64.7%) of women and 18/27 (66.7%) of men had a cholesterol of > 5mmol/L and 52/93 (55.9%) of women and 17/26 (65.4%) of men had an LDL of > 3 mmol/L. Full details of the lipid abnormalities are shown in the table 2.

**Table 2:** Prospective study of lipid profiles in patients with cholelithiasis

Lipids (abnormal range)	Females	Males	P value *
Cholesterol (> 5.0 mmol/L)	66/102 (64.7 %)	18/27 (66.7 %)	ns
Triglycerides (> 1.92 mmol/L)	37/102 (36.3 %)	7/27 (25.9 %)	ns
HDL cholesterol (< 1.00 mmol/L)	9/93 (9.7 %)	4/26 (15.4 %)	ns
LDL cholesterol (> 3.00 mmol/L)	52 / 93 (55.9 %)	17 / 26 (65.4 %)	ns

* Chi square with 1 degree of freedom. ns = not significant.

## Discussion

Unlike patients with renal stones, only a third of patients who had a cholecystectomy had a full lipid profile to identify an underlying metabolic disorder. These were done mostly by their GP’s. Although the initial retrospective study showed that this was abnormal in over 76% of women and 70% of men, this increased to 81% in women when studied prospectively. Hypercholesterolaemia was the most common abnormality in both sexes followed by a raised LDL cholesterol.

Hypercholesterolemia was statistically more significant among women in the retrospective group but this was not the case in the prospective group, probably because of fewer cases. A quarter of males and third of females also had hypertriglyceridaemia, an increasingly recognised risk factor for heart disease and diabetes[7, 17]. Men have a statistically significant lower HDL than women but this is less frequent, this was again observed only in the retrospective group.

We were unable to retrospectively analyse patients with cholelithiasis in this study, as our radiological database cannot provide this information. We overcame this problem by prospectively studying patients with gallstones although some did not have a complete profile. This would underestimate the true incidence. Although this study has only considered abnormal lipids, patients with gallstones are more likely to have additional factors for coronary heart disease, such as obesity, which would increase their risk. Using the current NSF guidelines for coronary heart disease it is likely that a large proportion of patients with gallstones should have treatment for their hyperlipidaemia in the presence of other risk factors.

From the UK cholecystectomy data, around 25,000 women and 6250 men are at an increased risk of developing coronary artery disease and stroke if not managed according to the NSF guidelines. If this risk is applied to all patients with gallstone disease, over 2.7 million women and nearly 690,000 men are at risk.

This study highlights one of the major weaknesses of current practice, namely that gallstones are regarded only as a surgical condition needing cholecystectomy. They should now be regarded as a marker of an underlying metabolic disorder and investigated and treated accordingly. Further studies of patients with gallstones detected on ultrasound will help establish the true incidence of the lipid abnormalities although this study shows it is already very high.

We recommend that all patients who have cholelithiasis should now have a full lipid profile, including HDL and LDL, as a routine part of their clinical assessment.
